# Sulfarotene Inhibits Colorectal Cancer via Mitigating Natural-Killer-Cell-Induced Stemness

**DOI:** 10.3390/ph17030387

**Published:** 2024-03-18

**Authors:** Keshu Hu, Yu Dong, Jiayu Zhang, Mengling Liu, Xun Sun, Xin Cao, Pengfei Zhang, Tianshu Liu

**Affiliations:** 1 Department of Medical Oncology, Zhongshan Hospital, Fudan University, Shanghai 200032, China; 2 Tianjin Medical University Cancer Institute and Hospital, Tianjin 300060, China; 3 Department of Medical Oncology, Shanghai Geriatric Medical Center, Shanghai 201104, China; 4 Cancer Center, Zhongshan Hospital, Fudan University, Shanghai 200032, China; 5 Center of Evidence-Based Medicine, Fudan University, Shanghai 200032, China

**Keywords:** colorectal cancer, natural killer cell, stemness, sulfarotene

## Abstract

Tumor cell stemness stands out as a pivotal factor driving tumor recurrence or metastasis and significantly contributes to the mortality of patients with colorectal cancer (CRC). Recent research has unveiled a link between immune-active cells and the induction of tumor cell stemness, ultimately leading to heightened resistance to treatment. In this study, stemness in CRC cell lines was assessed after co-culture with natural killer (NK) cells, both with and without sulfarotene administration. Furthermore, a CRC xenograft model was utilized to scrutinize the in vivo efficacy of sulfarotene in overcoming stemness induced by NK cell activation. As a result, CRC cells exhibited significant stemness after NK cell co-culture, as evidenced by the upregulation of several stemness markers associated with cancer stem cells. Moreover, these cells demonstrated remarkable resistance to commonly used chemotherapy agents for CRC, such as oxaliplatin and irinotecan. Importantly, sulfarotene effectively reversed the altered stemness of CRC cells in both in vitro and in vivo assays. In conclusion, sulfarotene emerges as a promising therapeutic strategy for overcoming colorectal cancer resistance to NK cells by effectively inhibiting stemness remodeling. This study underscores the potential of sulfarotene in augmenting NK-cell-mediated immune surveillance, proposing a novel immunotherapeutic approach against colorectal cancer.

## 1. Introduction

Colorectal cancer (CRC) ranks as the third most prevalent malignant neoplasm and stands as the second-leading cause of cancer-related mortality [1,2]. The elevated mortality associated with CRC can be attributed to tumor metastasis and recurrence [3,4]. Recently, immune checkpoint blockade (ICB) therapy has emerged as a groundbreaking approach in anti-tumor strategies [5]. However, the effectiveness of ICB, with or without cytotoxic T lymphocyte antigen 4 (CTLA4) inhibitors, remains limited to patients exhibiting high microsatellite instability (MSI-H) [6]. The majority of patients categorized as mismatch repair proficient (MMR-p) or microsatellite stable (MSS) are presumed to have lower immune activity, as indicated by a low or negative PD-L1 combined positive score (CPS), and generally exhibit poor responsiveness to ICB therapy [7].

While the role of T cells and tumor-associated macrophages (TAMs) has been extensively discussed in the context of ICB treatments [8,9], emerging evidence highlights the crucial involvement of innate immune cells, particularly natural killer (NK) cells, in this process [10]. Functioning as essential cytotoxic cells, NK cells recognize stressed cells and produce cytotoxic agents, such as granzyme, perforin, and interferon-γ (IFN-γ), to eliminate these cells. In doing so, they play a vital role in activating adaptive immune responses [11]. The absence of NK cells results in reduced cell death and antigen release from the neoplasm, leading to insufficient activation of the adaptive immune system. Consequently, this lack of activation ultimately results in a non-responsive state to ICB treatment [10]. Beyond the monoclonal antibodies (mAbs) targeting PD-(L)1, which is most frequently adopted so far, novel immune checkpoint molecules have been explored for possible clinical translation and better prognosis. T cell immune receptor with Ig and ITIM domains (TIGIT) has recently been discovered to express on T cells and NK cells and function as a strong immune-suppressive molecule [12]. TIGIT mAbs such as tiragolumab are currently undergoing several clinical trials, for instance the SKYSCRAPER series, which provides promising prospect of the novel ICB strategy [13].

Cancer stem cells (CSCs) are characterized by their stem-like properties, including the ability to self-renew and proliferate [14]. CSCs significantly contribute to the initiation of malignancies, treatment resistance, relapse, and metastasis of cancers. They often express stemness markers such as CD133, CD44, CD90, NANOG, and OCT4, with variations depending on their origin [15]. Studies have revealed that malignant cells exhibit high plasticity, allowing for the interconversion of CSCs and non-CSCs under specific circumstances [16]. Chemotherapies have been shown to reinforce cancer stemness, leading to the failure of such treatments [17,18]. While immune cells were traditionally considered to eliminate tumor cells more specifically and effectively compared to chemotherapy agents, a recent study indicated that IFN-γ produced by T cells enhanced a cancer stem cell signature in cancer patients treated with ICB therapy [19]. This suggests that the failure of immunotherapy might be linked to the enhancement of the stemness of tumor cells. Therefore, the question of whether the efficacy of ICB treatment can be improved by inhibiting CSCs remains unclear.

Despite the perceived resistance of CSCs to cytotoxic agents, they exhibit a notable vulnerability to drugs targeting stemness. One of the most well-known and widely used stemness-targeting drugs is all-trans retinoic acid (ATRA) [20]. In our previous studies, we identified a new synthetic retinoid, named sulfarotene (WYC-209), which effectively overcomes the stemness of hepatocellular carcinoma by suppressing the SOS2-RAS pathway [21]. Sulfarotene demonstrated potent and selective activity in suppressing the growth of CSC-like cells with negligible toxicity [22]. However, further investigation is required to determine whether sulfarotene can effectively curb the increase in the stemness of CRC induced by ICB therapy.

In the present study, we have observed a noteworthy increase in CSC-like features in CRC cells when exposed to activated NK cells. These stem-like CRCs exhibit resistance to cytotoxicity but show susceptibility to sulfarotene. Additionally, our data reveal that NK cells within the tumor microenvironment (TME) of CRC display inactivated phenotypes and exhausted signatures. In vivo experiments further demonstrate that while blocking TIGIT alone can transform NK cells from an incapacitated state to activation, it also leads to the enhancement of CSC phenotypes in CRC cells. Notably, this enhancement can be alleviated by the combination treatment of sulfarotene. Therefore, our study underscores sulfarotene as a potential therapeutic agent to ameliorate ICB-treatment-induced CSC-like progression in CRC, ultimately yielding satisfactory therapeutic effects.

## 2. Results

### 2.1. Co-Culturing with NK Cells Induces Stem-like Phenotypes of CRC Cells

**Figure 1:**
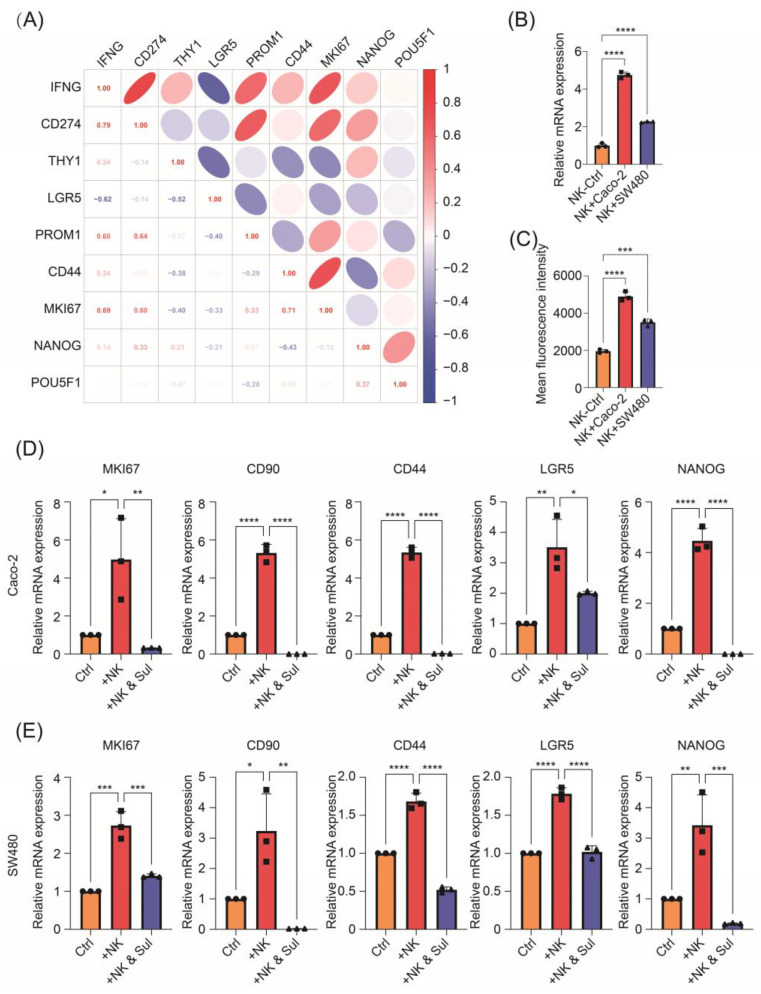
Stem-like features of CRC cells are induced by NK cells. (**A**) The correlation between stemness markers and PDL1, derived from tumor cells, and IFNG expression, derived from NK cells. (**B**,**C**) The mRNA level (**B**) and the fluorescence intensity (**C**) of IFN-γ in NK-92 co-cultured with Caco-2 or SW480 for 24 h. (**D**,**E**) The mRNA level of indicated stemness markers in Caco-2 or SW480 co-cultured with NK-92 for 24 h and further treated with or without 1 μM sulfarotene for 24 h. Data are presented as mean ± SD and are representative of at least three separate experiments. * *p* < 0.05, ** *p* < 0.01, *** *p* < 0.001, **** *p* < 0.0001.

As the main effector molecule of NK cells, IFN-γ has been proved to induce CSC plasticity in breast cancer, which contributes not only to chemotherapy and target therapy resistance but also to immunotherapy resistance [19]. Therefore, we first analyzed the correlation between IFNG expression level and stem-like state markers using single-cell sequencing analysis of untreated MSS CRC samples from a published dataset (GSE146771) [23]. As expected, positive correlations were found between IFNG expression level and CSC markers expression (e.g., THY1, CD44, MKI67, and NANOG) (Figure 1A). Besides several markers indicating stemness or proliferation, CD274 (encoding PD-L1) expression also demonstrated a remarkable coordinated variation (Figure 1A), which further validated the IFN-γ signature as the IFN-γ-STAT-PD-L1 pathway had already been well investigated previously [24]. 

To further validate the stem-like alteration in CRC cells, we conducted the co-culture system of CRC cells with NK-92 cells in vitro. NK cells produced a higher level of IFN-γ after being co-cultured with CRC cells (Figure 1B,C), indicating the activation of NK-92 cells after the co-culture. Meanwhile, co-culture with NK cells upregulated the expression of KI67, a sign of proliferation for tumor malignancy, and stemness markers CD90, CD44, LGR5, and NANOG in CRC cells (Figure 1D,E). Collectively, these data suggest that activated NK cells act as a strong driver of the CSC phenotype.

### 2.2. Stem-like Altered CRC Cells Demonstrate Cytotoxic Resistance but Vulnerability to Sulfarotene

**Figure 2:**
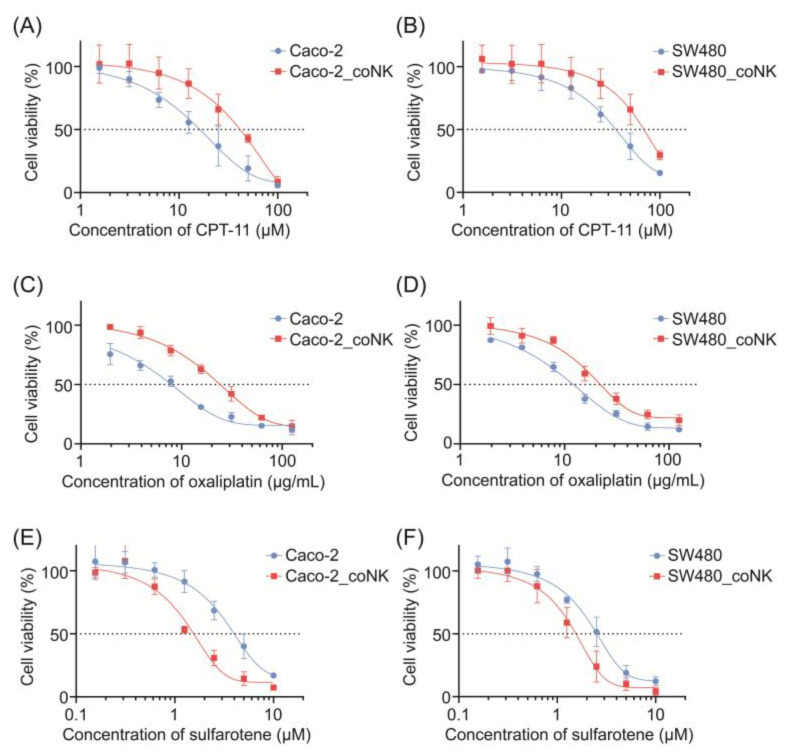
CRC cells with stem-like alterations show cytotoxic resistance but are susceptible to sulfarotene. (**A**–**F**) The CCK8 viability assays were used to measure CPT-11 (**A**,**B**), oxaliplatin (**C**,**D**), and sulfarotene (**E**,**F**) sensitivity changes at 48 h in Caco-2 (**A**,**C**,**E**) and SW480 (**B**,**D**,**F**), which were previously co-cultured with or without NK-92 for 24 h. The results were representative of three separate experiments. Data are presented as mean ± SD.

To comprehensively characterize CRC cells with elevated stemness, we detected their sensitivity alternations to chemotherapy reagent irinotecan (also known as CPT-11), oxaliplatin, and a synthetic retinoid sulfarotene (also known as WYC-209) which was previously reported to target and inhibit CSCs. As depicted in Figure 2A–F, CRC cells demonstrated increased resistance to chemotherapy but were more sensitive to sulfarotene after being co-cultured with NK cells. Furthermore, compared with being co-cultured with NK cells alone, significantly downregulated stemness properties with decreased expression of KI67, CD90, CD44, LGR5, and NANOG were observed in the co-cultured CRC cells after sulfarotene administration (Figure 1D,E).

**Figure 3:**
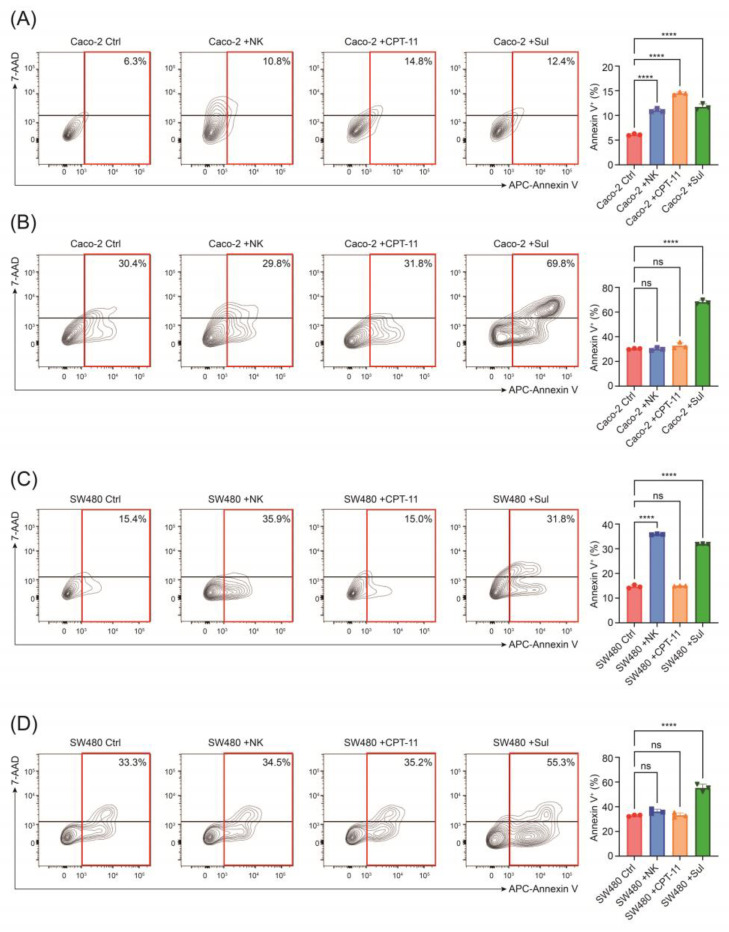
Pretreatment with NK-92 elevates CRC cells apoptosis. (**A**–**D**) Flow cytometric analysis and quantitation of apoptosis of Caco-2 (**A**,**B**) and SW480 (**C**,**D**) after co-culture with NK cells or drug administration for 24 h, pretreated with (**B**,**D**) or without (**A**,**C**) NK-92 for 24 h. Representative flow cytometry plot is from three independent experiments. Data are presented as mean ± SD. ns, not significant; **** *p* < 0.0001.

Moreover, we conducted apoptosis analysis to further evaluate the cytotoxic effect of CPT-11, sulfarotene, and NK cells in CRC cells pretreated with or without NK cells. FCM analysis showed that CPT-11 and NK cells enhanced apoptosis of Caco-2 and SW480, respectively. However, NK cells pretreatment increased cytotoxic resistance in CRC cells, while it promoted cell apoptosis induced by sulfarotene (Figure 3A–D). These data indicate that the co-cultured CRC cells showed stem-like characteristics, which could be targeted and reversed by the synthetic retinoid sulfarotene.

### 2.3. Different Gene Expression Profile between NK Cells from Periphery and MSS-Type CRC

**Figure 4:**
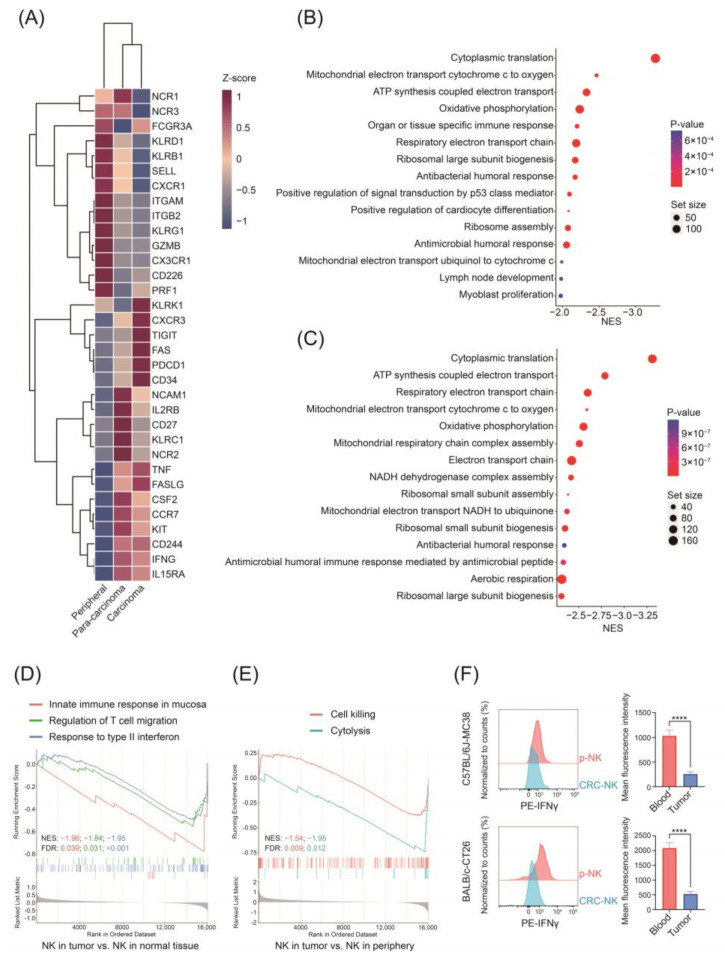
CRC-derived NK cells demonstrate significant immunosuppression. (**A**) The heatmap of functional NK markers on NK cells derived from periphery, normal colon tissue, and CRC tissue. (**B**,**C**) Gene ontology (GO) analysis was conducted to identify the top 15 pathways between NK cells from CRC tissue and normal colon tissue (**B**), and NK cells from CRC tissue and periphery (**C**). (**D**) Gene set enrichment of NK cells derived from CRC tissue and normal colon tissue. (**E**) Gene set enrichment of NK cells derived from CRC tissue and periphery. (**F**) Representative plots and quantification of IFN-γ in pNK cells and NK cells isolated from the subcutaneous tumor (n = 5 for each group). Data are presented as mean ± SD. **** *p* < 0.0001.

MSS-type CRC was mostly considered as immune-excluded or immune-desert tumors and responded poorly to immunotherapy, in which neither innate nor adaptive immune response might be adequately triggered. Therefore, to characterize NK cells in MSS CRC, we first analyzed the single-cell RNA-sequencing data of untreated MSS CRC samples from a published dataset (GSE146771). We re-classified the NK cells by their location, whether they were in peripheral blood, normal colon tissue, or CRC tissue. In NK cells derived from the CRC tissue, genes associated with cytotoxic features such as FCGR3A (also known as CD16) and PRF1 (encoding perforin) decreased, and inhibitory markers such as TIGIT, FAS, and PDCD1 (also known as PD-1) increased (Figure 4A). We then conducted a gene ontology (GO) analysis to decipher the functional characteristics of NK cells in patients with CRC. As shown in Figure 4B,C, NK cells from the CRC tumor micro-environment manifested a significant downregulation in several metabolic sets, such as oxidative phosphorylation, ATP synthesis, and respiratory electron transport chain, which was associated with decreased function of NK cells [25]. Gene set enrichment analysis (GSEA) also revealed that compared with NK cells derived from the normal colon tissue, CRC-NK cells were less enriched in mucosa immune responses and activation of adaptive immune responses (Figure 4D). Additionally, CRC-NK cells had downregulated function in cytotoxicity compared with peripheral NK (pNK) cells (Figure 4E). Thus, these sequencing results suggest NK cells with dysfunctional status infiltrated the tumor microenvironment (TME) of CRC.

To further validate the results from single-cell profiling analysis, BALB/c or C57BL/6J mice subcutaneously injected with CRC cell line CT26.WT or MC38 were established, respectively. We firstly isolated primary cells from peripheral blood and tumor tissue and performed FCM to detect the function of NK cells. We found that compared with pNK cells, NK cells derived from tumor tissues possessed remarkably lower IFN-γ levels, on behalf of the function of cytotoxicity (Figure 4F). Collectively, these data demonstrate that NK cells in TME exhibited immunosuppressive phenotypes and exhausted signatures that are conducive to an immune-tolerant TME.

### 2.4. Sulfarotene Further Augments the Effect of Anti-TIGIT Immunotherapy In Vivo

**Figure 5:**
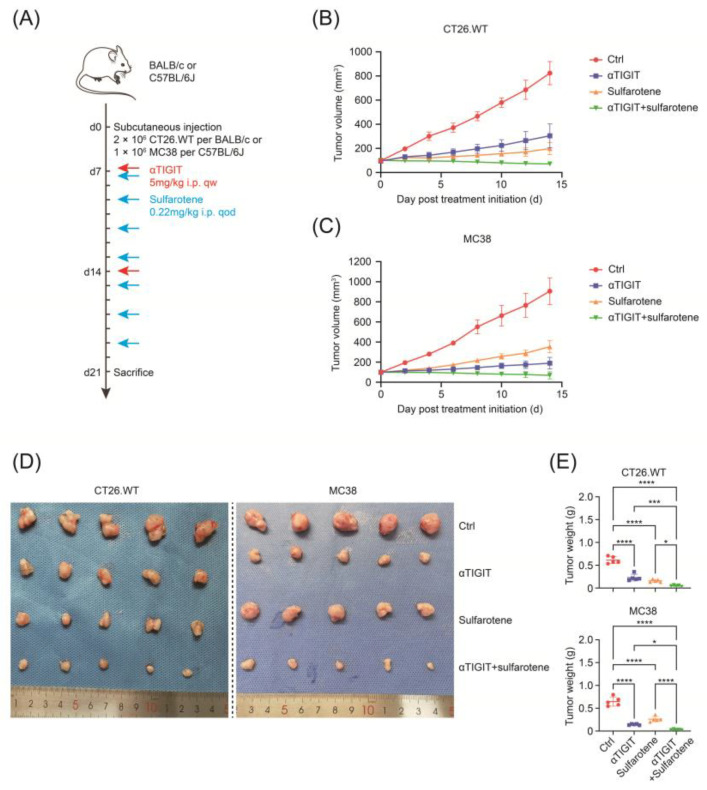
Combination of anti-TIGIT immunotherapy and sulfarotene achieves preferable efficacy. (**A**) Schematic illustration of TIGIT blocking antibody and sulfarotene treatment in mice subcutaneously injected with CT26.WT or MC38. (**B**,**C**) The growth curves of the subcutaneous tumors in mice subcutaneously injected with CT26.WT (**B**) or MC38 (**C**) treated as indicated. (**D**,**E**) The images (**D**) and quantification (**E**) of the subcutaneous tumors resected from mice subcutaneously injected with CT26.WT or MC38 treated as indicated. n = 5 for each group. Data are presented as mean ± SD. * *p* < 0.05, *** *p* < 0.001, **** *p* < 0.0001.

As MMR-p CRC is considered resistant to anti-PD-1/PD-L1 monotherapy, previous research has revealed that TIGIT was associated with NK cell exhaustion, rather than PD-1 molecules. Thus, the effects of sulfarotene and anti-TIGIT immunotherapy on the growth of subcutaneous xenograft nodes derived from CT26 and MC38 in vivo in BALB/c or C57BL/6J mice were investigated. As depicted in Figure 5A, after tumors were palpable, mice were given anti-TIGIT (5 mg/kg, i.p, qw) and sulfarotene (0.22 mg/kg, i.p, qod), alone or combined. The results showed that both the volumes and weights of formed CRC tumor nodes were reduced by anti-TIGIT or sulfarotene, which could be further augmented by the combination of treatment with anti-TIGIT and sulfarotene (Figure 5B–E).

### 2.5. Sulfarotene Reverse Stemness Alteration Induced by Unleashed NK Activity

**Figure 6:**
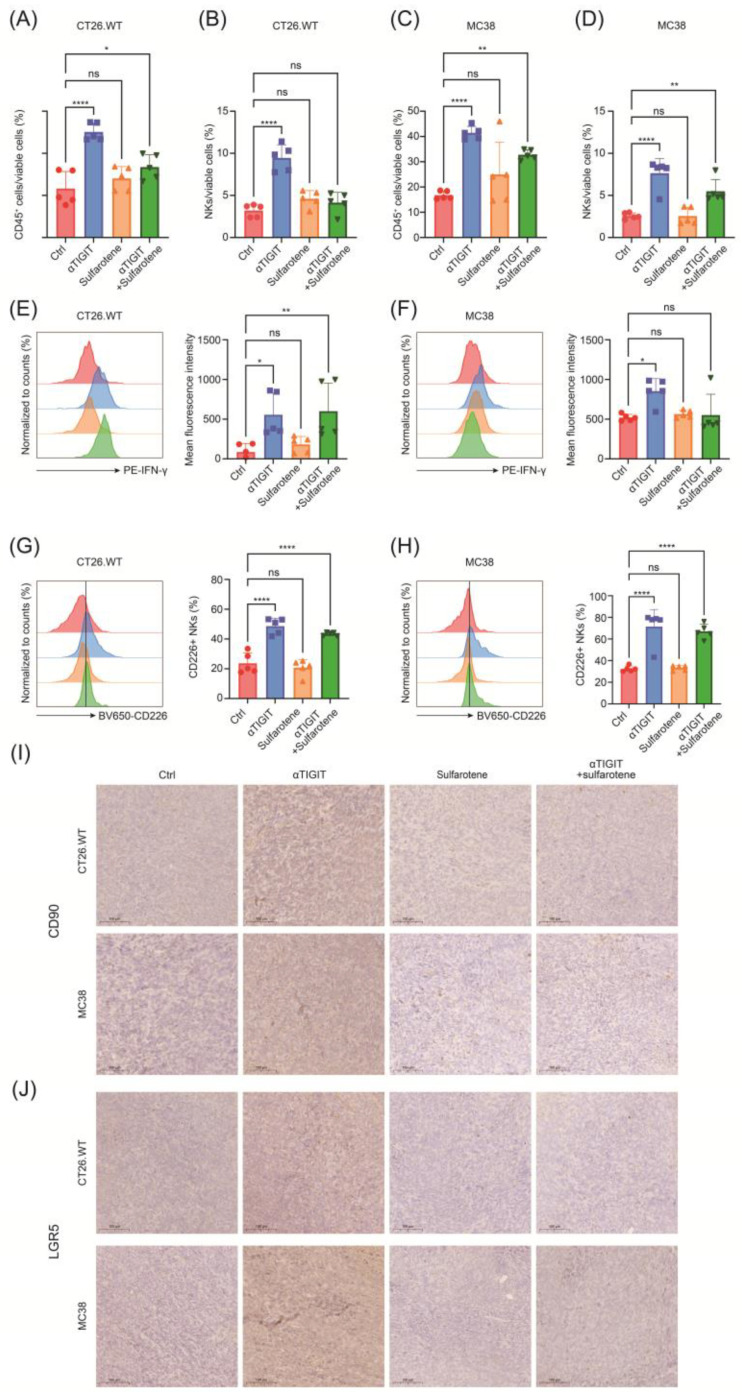
Sulfarotene alleviates TIGIT blockage-induced stemness conversion of CRC cells. (**A**–**D**) The flow cytometric analysis of the frequency of total CD45+ immune cells and NK cells infiltrated into the subcutaneous tumor treated as indicated. (**E**,**F**) Quantification of IFN-γ of tumor-infiltrating NK cells from CT26.WT (**E**) or MC38 (**F**) treated as indicated. (**G**,**H**) Frequency of cells expressing CD226 among tumor-infiltrating NK cells from CT26.WT (**G**) or MC38 (**H**) treated as indicated. (**I**,**J**) Representative immunohistochemical staining images showing the expression of stemness markers in tissues from CT26.WT (**I**) or MC38 (**J**) treated as indicated. The color area of the flow cytometry plot refers to the color of the column on the histogram to its right. n = 5 for each group. Data are presented as mean ± SD. ns, not significant; * *p* < 0.05, ** *p* < 0.01, **** *p* < 0.0001.

Next, we investigated the alternations of the phenotype and function of NK cells in CRC treated with or without anti-TIGIT and sulfarotene, alone or combined. As a result, anti-TIGIT treatment increased the infiltration of immune cells and NK cells (Figure 6A–D). It also significantly enhanced the function of NK cells manifested as augmented the frequency of CD226 as well as the production of IFN-γ (Figure 6E–H). Thus, blocking TIGIT contributed to NK cell activation in the CRC subcutaneous tumor. However, sulfarotene treatment might have no direct impact on the numbers or the functions of NK cells in TME.

Given the function of sulfarotene in inhibiting tumor stemness, immunohistochemical staining was conducted to assess the stemness alterations of CRC treated as above. Of note, anti-TIGIT immunotherapy prominently increased the CD90 and LGR5 expression in the tumor, which was markedly reversed by the addition of sulfarotene (Figure 6I,J). Taken together, these findings reveal effectiveness and the rationale of using sulfarotene in combination with immunotherapy, which inhibits the stemness remodeling and contributes to CRC regression.

## 3. Discussion

Anti-tumor therapy has entered the era of immunotherapy. Immune checkpoint inhibitors (ICIs), especially programmed cell death protein 1 (PD-1) monoclonal antibody, are approved for use in a variety of tumors, while they had less effects in patients with MSS-CRC [26]. Our study demonstrated that NK cells in TME of MSS-type CRC demonstrate an immunosuppressive phenotype, while activated NK cells, characterized by elevated production of IFN-γ, contributed to remarkable upregulation of CRC stemness. These CSC-like CRC cells were less sensitive to chemotherapeutic drugs, while vulnerable to sulfarotene. Combination of treatment with blockage of TIGIT and administration of sulfarotene in CRC mouse models resulted in activated NK cells with higher production of IFN-γ and decreased stemness signatures of tumor cells. Our results indicate that sulfarotene, as a stemness-targeting therapy, helps ICB to achieve better anti-tumor effects.

The importance of T cell immune response has been well introduced in anti-tumor immune therapies, which aim to blockade the inhibitory ligands and sufficiently unleash the T cell activity. Anti-PD-1 or anti-PD-L1 strategies have obtained clinical achievements in several types of malignancies, and the quantitative evaluation of tumor PD-L1 expression has been considered as a significant prognostic factor. Although immunotherapy has shown good results in upper gastrointestinal tumors [27], in MMR-p CRC, immunotherapy has brought little effect with only a 0–2% overall response rate [26]. It was reported that the expression level of PD-L1 was positively correlated with the efficacy of immunotherapy [28]. However, PD-L1 demonstrates mostly low expression accordantly in patients with MMR-p CRC [29]. As PD-L1 is upregulated subsequent to immune activation and IFN-γ releasing, its low expressions validate that the anti-tumor immunity is not adequately activated in the TME of MMR-p CRCs [28]. Therefore, we asked whether the function of NK cells was operational, for their duties as innate immune system were indispensable for valid acquired immune responses [30]. Through re-analyzing a published single-cell sequencing dataset, we revealed that NK cells in the TME of MMR-p CRC patients exhibited a significant immunotolerant phenotype and were functionally inhibited, which might be a potential cause of the inactive immune response to ICB.

Limited preclinical studies have explored the potential of boosting NK cell–mediated anti-tumor immunity. A recent study found that PD-1+ NK cells demonstrated an activated phenotype and did not mark NK cells with an exhausted phenotype [10]. As a co-inhibitory receptor, TIGIT is considered to contribute to immunotolerance by restraining not only immune responses mediated by T cells [31,32] but also those regulated by NK cells via binding with its ligand, CD155, on target cells [33,34]. Zhang et al. discovered that in a mouse model of subcutaneously administered CT26 colon cancer, upregulation of TIGIT on tumor-infiltrating NK cells was observed, but only a low frequency of these cells with surface expression of PD-1 (less than 10%) [30]. TIGIT+ NK cells exhibited lower killing activity against CD155+ MHC class-I deficient melanoma FO-I as compared with TIGIT− NK cells [34]. Blockade of TIGIT could prevent NK cell exhaustion and enhance the synthesis of IFN-γ by NK cells in tumor-bearing mice [30,34,35]. Here, we adopted the mouse TIGIT antibody (mu10A7) as the in vivo strategy for NK activation [36]. In both CT26- and MC38-beared mouse models, blocking TIGIT significantly suppressed tumor growth. The therapeutic effects were accompanied by a higher frequency of tumor-infiltrating NK cells, a reversed exhaustion of NK cells, and an enhanced NK-cell-mediated anti-tumor immunity. Thus, NK cells are critical for the therapeutic effects of the blockade of TIGIT.

In line with a previous study, we also found that promoting the function of tumor immune cells would simultaneously increase the stemness of tumor cells. The remarkable self-renewal capacity and high tumorigenicity of resident CSCs are thought to account for the high recurrence rate, acquired drug resistance, poor prognosis, and treatment failure [37,38,39]. Both natural and synthetic trans-retinoic acid (RA) were applied to the hematopoietic system, particularly leukemia; nevertheless, efficacy against solid tumors remains inferior attributed to drug resistance, poor solubility, and short half-life [22]. Sulfarotene, initially discovered by retinoid library screening, demonstrates remarkable inhibition of human tumor-repopulating cells (TRCs), which possess significant stem-like characteristics. Comparing to all-trans-RA (ATRA), the synthetic retinoid greatly improved its physical property with high solubility and exhibited low-toxicity to non-cancerous cells or immune-competent mice, which endowed it to be more appropriate for possible clinical translation. Chen et al. found that sulfarotene could overcome stemness and thus contribute to metastasis or relapse elimination [22]. In terms of mechanism, sulfarotene interacts with retinoic acid receptors (RARs) and has been found to inhibit the SOS2 pathway, WNT pathway, or regulate epigenetic modifications in various malignancies [21,40,41]. Its molecular mechanisms in CRC or TME still need detailed interpretation.

In our study, sulfarotene effectively targeted stemness-enhanced CRC cells induced by IFN-γ, which was less sensitive to chemotherapy. Although Laurent Beziaud et al. reported that the addition of gabapentin, as an inhibitor for the stemness transition, could also prevent the IFN-γ-mediated induction of CSCs, sulfarotene was of anti-tumor capacity in a dose-dependent manner itself. In addition, our in vitro assays validated that sulfarotene could overcome the stemness even after the induction by IFN-γ, while gabapentin was administered simultaneously with IFN-γ, and its effect on stemness cells remains to be determined. Of importance, we observed that administration of sulfarotene had no impact on the frequency and functions of NK cells but improved the stemness shift of CRCs induced by TIGIT blockage and NK activation, highlighting the treatment efficacy of sulfarotene combined with immunological therapy in MMR-p CRC. We proposed that this stemness-targeting strategy would possess promising clinical translational value in synergy with immunotherapy, particularly with anti-TIGIT mAbs.

This study had several limitations. Further multi-omics studies are necessary to reveal how sulfarotene contributes to the whole TME, and still CRC patient TME and tumor alterations after ICB treatment are urgently needed to be investigated. The effectiveness of the combined strategy for human samples also requires further validation.

## 4. Materials and Methods

### 4.1. Data Availability and scRNA-seq Analyses

The scRNA-seq data were obtained from the GEO dataset GSE146771. The annotations of the cells remained as in the original text [23]. The analyses were conducted in the R studio. The differentially expressed genes were identified by the Seurat package, and the heatmap was drawn by the pheatmap package. Further GSEA analyses and visualizations were accomplished using the clusterProfiler, enrichplot, and ggplot2 packages.

### 4.2. Mouse Model

In the present study, 6- to 8-week-old male C57BL/6 or BALB/c mice were purchased from Shanghai JieSiJie Laboratory Animal Co, Ltd. (Shanghai, China). MC38 or CT26.WT cells were injected into the right flank of the mice at a total number of 1 × 10^6^ or 2 × 10^6^ per mouse, respectively. One week after inoculation, the mice were randomly grouped into different treatment groups or vehicle/IgG control. The tumor sizes were evaluated every 2 days referring to the following formula: 0.5 × length × width × width (mm^3^). For drug treatments, anti-TIGIT or IgG isotype antibodies were administered 5 mg/kg intraperitoneally (i.p) weekly, and sulfarotene was 0.22 mg/kg i.p every other day. The mice were sacrificed after 2 weeks of treatment, and the tumors were resected for analyses.

### 4.3. Cell Lines and Cell Culture

The human CRC cell lines Caco-2 and SW480, the murine CRC cell lines CT26.WT and MC38, and the human NK cell line NK-92.MI were purchased from American Type Culture Collection (ATCC, Manassas, VA, USA). The cells were cultured in accordance with the ATCC instructions. In the co-culture assays of human CRC cells and NK-92.MI, 5 × 10^4^ CRC cells were seeded into a 12-well plate 1 day prior to NK co-culture. The NK cells had been labeled with CFSE before being co-cultured with the CRC cells, in order to be well distinguished by the flow cytometry analyses afterwards. In total, 2.5 × 10^4^ NK cells were used for co-culture with the CRC cells in each well upon an effector–target ratio of 1:2. After 24 h of co-culture, the NK cells in the supernatant were removed. The co-cultured CRC cells were collected for direct detection or counted and re-seeded to plate for further assays. For the in vitro assays except CCK-8, sulfarotene was administered at a final concentration of 1 μM for 24 h, and CPT-11 of 10 μM for 24 h as well.

### 4.4. CCK-8 Assays

The CRC cells were seeded 5 × 10^3^ per well into the 96-well plate 24 h before drug administration. After being treated at for 48 h, 10 μL CCK-8 reagents were added to each well including the blank. The absorptions at 450 nm wavelength were measured after 2 h, and the cell viabilities were calculated referring to the control well.

### 4.5. Single-Cell Suspension Preparation

The subcutaneous tumors were digested into a single-cell suspension before flow cytometry referring to previous study [23]. Briefly, the tumors were placed in the RPMI-1640 medium containing collagen D (0.5 mg/mL, Roche, Basel, Switzerland) and DNase I (40 U/mL, Roche, Switzerland) before being disrupted by the gentleMACS Dissociator (Miltenyi Biotech, Bergisch Gladbach, Germany). The suspension was then incubated at 37 °C while gently shaking at 150 rpm for 15 min and went through the disrupting program once further. Finally, the single-cell suspension was obtained by passing through a 70 μm filter. The cells were washed and resuspended in PBS before flow cytometry staining.

### 4.6. Flow Cytometry

For the staining of cells from mouse tumors, the cells were incubated with surface staining including anti-CD45, Fixable Viability Dye, anti-NKp46, anti-CD3, anti-CD226, and anti-TIGIT (Biolegend, San Diego, CA, USA). Next, the cells were fixed and permeabilized according to the kit instructions (BD Biosciences, San Jose, CA, USA). Intracellular IFN-γ was stained after permeabilization. The apoptosis staining was performed according to the kit instruction (Biolegend, USA). The labeled cells were washed and eventually detected by CytoFLEX (Beckman Coulter, Brea, CA, USA), and the data were analyzed using FlowJo software (Version 10.8.1).

### 4.7. qPCR

Total RNA from the cells was extracted using RNA-Quick Purification Kit (EZBioscience, Suzhou, China) according to the kit instructions. The reverse transcription and qPCR were conducted using reverse transcription mix and 2× SYBR green mix (Yeasen, Shanghai, China). The primer sequences in the assays are listed in Table 1.

### 4.8. IHC Staining

Briefly, the paraffin sections were deparaffinized and rehydrated. After antigen retrieval and blocking the endogenous peroxidase activity, the sections were blocked by goat serum and subsequently stained with primary antibody against CD90 or LGR5 (Affinity, San Francisco, CA, USA) overnight at 4 °C. The sections were pictured under microscopy after secondary antibody staining, DAB incubation, and hematoxylin counterstaining.

### 4.9. Statistical Analysis

The results are shown as the means ± SD. Hypothesis tests were performed by Student’s t tests between 2 groups or by ANOVA analyses among multiple groups. All statistical analyses were performed using GraphPad Prism 10. A *p*-value < 0.05 was considered statistically significant.

## 5. Conclusions

Collectively, our study has identified sulfarotene as a potentially effective agent that overcomes the stemness remodeling and NK cell resistance in CRC and highlights the importance of stemness-targeted treatment combined with ICB therapy for tumor control.

## Figures and Tables

**Table 1:** Primers used for qPCR in the study.

Gene	Forward Primer 5′-3′	Reverse Primer 5′-3′
ACTB	CATGTACGTTGCTATCCAGGC	CTCCTTAATGTCACGCACGAT
IFNG	TCGGTAACTGACTTGAATGTCCA	TCGCTTCCCTGTTTTAGCTGC
MKI67	AGAAGAAGTGGTGCTTCGGAA	AGAAGAAGTGGTGCTTCGGAA
THY1	ATGAAGGTCCTCTACTTATCCGC	GCACTGTGACGTTCTGGGA
CD44	CTGCCGCTTTGCAGGTGTA	CATTGTGGGCAAGGTGCTATT
LGR5	ATGTCGAAGCCCCATAGTGAA	TGGGTGGTGAATCAATGTCCA
NANOG	AAGGTCCCGGTCAAGAAACAG	CTTCTGCGTCACACCATTGC

## Data Availability

Data is contained within the article.
